# *Ex Vivo* Chemosensitivity Profiling of Acute Myeloid Leukemia and Its Correlation With Clinical Response and Outcome to Chemotherapy

**DOI:** 10.3389/fonc.2021.793773

**Published:** 2022-01-05

**Authors:** Yi Zhang, Min Ji, Jin-Yan Zhao, Hua-Feng Wang, Chong-Wu Wang, Wei Li, Jing-Jing Ye, Fei Lu, Li-Hui Lin, Yan-Ting Gao, Jie Jin, Li Li, Chun-Yan Ji, Joan Ballesteros, Hong-Hu Zhu

**Affiliations:** ^1^ Department of Hematology, The First Affiliated Hospital, College of Medicine, Zhejiang University, Hangzhou, China; ^2^ Zhejiang Province Key Laboratory of Hematology Oncology Diagnosis and Treatment, Hangzhou, China; ^3^ Zhejiang University Cancer Center, Hangzhou, China; ^4^ Department of Hematology, Qilu Hospital, Shandong University, Jinan, China; ^5^ Department of Laboratory Medicine, Shanghai General Hospital, Shanghai Jiao Tong University School of Medicine, Shanghai, China; ^6^ Zhejiang Laboratory for Systems & Precision Medicine, Zhejiang University Medical Center, Hangzhou, China; ^7^ R & D Department, Hosea Medical Technology (Beijing) Co., Ltd., Beijing, China; ^8^ R & D Department, Vivia Biotech, Madrid, Spain

**Keywords:** acute myeloid leukemia, *ex vivo* drug sensitivity test, chemosensitivity, precision medicine, complete remission

## Abstract

We evaluated the predictive value of the *ex-vivo* PharmaFlow PM platform in measuring the pharmacological activity of drug combinations consisting of 20 different chemotherapy regimens (20 Tx) administered in 104 acute myeloid leukemia (AML) patients. The predicted sensitivities of alternative treatments for each patient were ranked in five 20% categories, from resistant to sensitive (Groups 1–5). The complete remission (CR) rates of the five groups were 0%, 12.5%, 38.5%, 50.0%, and 81.3%, respectively. The heat map showed a good relationship between drug sensitivity with CR (Group 4 + 5 vs. Group 1 + 2+3: 77.5% vs. 27.3%, p = 0.002) and the European Leukemia Net risk group (22.6% vs. 63.6%, p = 0.015). The predicted coincidence rate was 90.9% in Group 1 + 2 and 81.3% in Group 5. According to the recommendations of the PharmaFlow PM platform, the CR rate would have increased by about 16.3% in one cycle. The overall survival (OS) was shorter in patients predicted to be resistant (Group 1 + 2 vs. Group 3 + 4+5, p = 0.086). In multivariable analysis, CR after one cycle was an independent prognostic factor for OS [p = 0.001; 95% CI 0.202 (0.080–0.511)], and *ex-vivo* chemosensitivity was a potential predictive factor for OS [p = 0.078; 95% CI 0.696 (0.465–1.041)]. To conclude, the PharmaFlow PM platform is a rapid and valuable tool for predicting clinical response and outcomes in AML patients.

## Introduction

Acute myeloid leukemia (AML) is a heterogeneous hematological malignancy and is the most common type of adult leukemia. About 60% of newly diagnosed AML patients receiving frontline therapy attain a complete remission (CR), yet 30%–40% of patients experience relapse (1, 2). Treatment for relapsed patients is difficult and limited (3). Refractoriness to induction therapy and relapse after CR are still the most challenging aspects in AML. The 5-year overall survival (OS) of AML patients aged younger than and older than 60 years is about 30%–40% and less than 15%, respectively (4, 5). Currently, the diagnosis of AML is based on the morphology, immunology, cytogenetics, and molecular biology (MICM) characteristics, treatment regimens often made based on the doctors’ experience. How to formulate individualized treatment plans for patients based on multiple drug combinations is the unmet need of AML treatment.

In recent years, with the development of precision medicine, our understanding of the molecular mechanisms and prognosis of AML has improved substantially (6, 7). However, technologies that help in doctors’ rapid and accurate treatment decisions among heterogeneous patients are still limited. The *in-vivo* chemosensitivity method for constructing the patient-derived xenograft (PDX) mouse model is complicated, time-consuming, and expensive. The *in-vitro* single-cell culture methods such as 3-(4,5-dimethylthiazol-2-yl)-2,5-diphenyltetrazolium bromide assay and ATP assay cannot distinguish between tumor cells and non-tumor cells, or simulate the *in-vivo* microenvironment (8–10). Nowadays, high-throughput *ex-vivo* drug sensitivity screening test is used to detect pharmacological activity in the treatment of AML (11–18). Herein, we tested the prediction value of the PharmaFlow platform using bone marrow samples of 104 AML patients.

## Materials and Methods

### Patients

Between September 2017 and June 2020, a total of 104 patients aged 13–64 years who were diagnosed with AML were enrolled from 3 different Chinese medical centers (The First Affiliated Hospital, College of Medicine, Zhejiang University, Qilu Hospital of Shandong University, and Shanghai General Hospital). Patients with acute promyelocytic leukemia (APL), secondary to myelodysplastic syndromes and therapy-related AML, were excluded. The diagnostic and classification criteria were based on the 2016 World Health Organization (WHO) (19) and 2017 European Leukemia Net (ELN) criteria (20). All patients provided their informed consent for participation in the study. The study protocol followed the Declaration of Helsinki, which was also approved by the Research Ethics Committee of The First Affiliated Hospital, College of Medicine, Zhejiang University, Qilu Hospital of Shandong University, and Shanghai General Hospital.

### Chemotherapy Regimens

All treatments were administered during the regular clinical practice, without taking into consideration the PharmaFlow PM test results. Induction regimen options were based on the age and physical condition of the patient. Aggressive induction chemotherapy regimen such as DA, IA, and HAA was applied to young and fit patients (age <60), while the relatively less aggressive chemotherapy regimens such as CAG and AA were applied to elderly or unfit patients, and regimens like FLAG and MA were used for salvage treatment.

The main treatment options include DA: daunorubicin (60 mg/m^2^ d:1–3) + cytarabine (100 mg/m^2^ d:1–7), IA: demethoxydaunor (10 mg/m^2^ d:1–3) + cytarabine (100 mg/m^2^ d:1–7), HAA: homoharringtonine (2 mg/m^2^ d:1–7) + cytarabine (100 mg/m^2^ d:1–7) + aclacinomycin (12 mg/m^2^ d:1–7), CAG: cytarabine (10 mg/m^2^ q12h d:1–14 or 20 mg/m^2^ q12h d:1–7) + aclarubicin (12 mg/m^2^ d:1–4) + granulocyte colony-stimulating factor (G-CSF, 200 mg/m^2^ qd until neutrophil recovery), AA: aclacinomycin (12 mg/m^2^ d:1–5) + cytarabine (100 mg/m^2^ d:1–5), FLAG: fludarabine (30 mg/m^2^ d:1–5) + cytarabine (1–2 g/m^2^ d:1–5), and MA: mitoxantrone (12 mg/m^2^ d:1–3) + cytarabine (100 mg/m^2^ d:1–7).

### Data Collection and Study Endpoints

Data were collected and double-checked by clinicians in each hospital, through case reviews, outpatient clinic records, and telephonic conversations. The last follow-up time was performed in September 2020.

The measured outcomes were CR and overall survival (OS). CR was defined as <5% bone marrow blasts and normal maturation of all cell lineages, no blast in the blood, absolute neutrophil count ≥1.0 × 10E^9^/L, platelet count ≥100 × 10E^9^/L, and no extramedullary leukemia. Relapse was defined as the reappearance of blasts in the bone marrow (>5%) or extramedullary leukemia in patients with previously documented CR. The refractory disease was defined as the failure to achieve CR after two cycles of standard induction therapies. OS was defined as the time from the first treatment to death, which was censored at the time of hematopoietic stem cell transplantation or the last follow-up.

### PharmaFlow PM Test

The PharmaFlow PM test was performed in the laboratories of the Hosea Precision Medicine (Beijing, China). Bone marrow samples were collected from patients at the time of diagnosis or relapse–refractory. Whole bone-marrow samples maintaining their native environment were incubated for 48 or 72 h in well plates containing 20 treatments representing the most common drugs and drug combinations used in induction treatments for AML patients (**Supplement 1**). The PM test activity was calculated by the percentage of the maximum area under the curve (AUC) from curve function models fitted to the results of the dose–response experiment. In addition, the test includes a measurement of synergy in drug combinations estimated by the application of surface interaction models. For each drug, a dose–response curve was measured with 8 concentrations covering a wide range of activity. Parameters AUC and EC50 were calculated from fitting these dose–response curves. The details of the working process and methods of the PharmaFlow PM test have been discussed in our publication (11, 21). The predicted sensitivities of alternative treatments for an individual patient are ranked in five 20% categories, ranging from most resistant 0%–19% (resistant, Group 1), 20%–39% (relatively resistant, Group 2), 40%–59% (intermediate, Group 3), and 60%–79% (relatively sensitive, Group 4) to most-sensitive 80%–100% (sensitive, Group 5).

### Statistical Analyses

Data were analyzed and visualized, and statistical comparisons were performed with the SPSS 25. p < 0.05 was considered to indicate statistical significance. Continuous variables were analyzed by the *Mann–Whitney U-test*, and categorical parameters were compared by *Pearson χ2* test or *Fisher’s exact test*. OS probabilities were estimated by the Kaplan–Meier survival analysis, and the differences in the survival curves were compared by the *log-rank test*. The hazard ratio of OS was estimated by using multivariate Cox hazard models. Univariate analyses included the following: white blood cells, platelet, age, BM blasts, CR after the first cycle (yes/no), induction treatments, ELN risk, and *ex vivo* drug sensitivity. Factors with p < 0.2 were applied in multivariate analyses.

## Results

### Patient Characteristics

A total of 104 patients were included, 51.9% (54/104) were male, and the median age was 43.5 years (range 13–64). The most frequently mutated genes in this series were fms-like tyrosine kinase 3 (*FLT3*) (29.8% of patients), Tet methylcytosine dioxygenase 2 (*TET2*) (28.8%), nucleophosmin 1 (*NPM1*) (24.0%), Wilms’ tumor suppressor gene1 (*WT1*) (22.1%), GATA binding protein 2 (*GATA2*) (19.2%), DNA methyltransferase 3 alpha (*DNMT3A*) (17.3%), neuroblastoma RAS viral oncogene homolog (*NRAS*) (14.4%), and isocitrate dehydrogenase 2 (*IDH2*) (13.5%). The ELN 2017 results were obtained in all patients; favorable and adverse karyotypes were found in 33.6% (35/104) and 26.9% (28/104) patients, respectively. Within this cohort, 94 patients were *de novo* and the other 10 patients were relapse–refractory. The detailed clinical data of gender, age, white blood cells and blast cell counts, AML French–American–British classification type, risk stratification, and induction treatment are summarized in **Table 1**.

**Table 1:** General characteristics of 104 AML patients.

Group	AML	Relapse
Cases	94	10
Gender (F/M)	44/50	6/4
Age, years	43.5 (13–64)	42.5 (27–60)
WBC, 10^9^/L	16.3 (0.15–305.3)	11.2 (1.8–55.5)
HB, g/L	83 (32–162)	80 (38–148)
PLT, 10^9^/L	36 (6–639)	15.5 (5–76)
Blasts, %	74 (18–95)	46 (22–96)
FAB		
M0	2 (2.1%)	0 (0.0%)
M1	6 (6.4%)	0 (0.0%)
M2	24 (25.5%)	4 (40.0%)
M4	23 (24.5%)	3 (30.0%)
M5	38 (40.4%)	3 (30.0%)
M6	1 (1.1%)	0 (0.0%)
ELN 2017		
Favorable	32 (34.0%)	3 (30.0%)
Intermediate	37 (39.2%)	4 (40.0%)
Adverse	25 (26.6%)	3 (30.0%)
CR/NR	74.5%/25.5%	50%/50%
CR after 1 cycle	57.4%	20%
Induction regimen		
IA	51 (54.3%)	5 (50.0%)
DA	30 (31.9%)	4 (40.0%)
sOther*^a^*	13 (13.8%)	1 (10.0%)
HSCT	23 (24.5%)	/
Chemosensitive group*^b^*		
1 (0–19)	1 (1.1%)	0 (0.0%)
2 (20–39)	3 (3.2%)	1 (10.0%))
3 (40–59)	5 (5.3%)	1 (10.0%)
4 (60–79)	16 (17.0%)	3 (30.0%)
5 (80–100)	69 (73.4%)	5 (50.0%)

WBC, white blood cell count; Hb, hemoglobin; PLT, platelet count; FAB, morphology according to French–American–British classification; ELN, European Leukemia Net; CR, complete remission; IA, idarubicin + cytarabine; DA, daunorubicin + cytarabine; HSCT, hematopoietic stem cell transplantation.

^a^HAA, MA, FLAG, low-dose cytarabine.

^b^Groups 1 to 5 were according to the optimal PharmaFlow results (highest score of the 20 treatments).

All 94 *de novo* AML patients were treated with chemotherapy as induction therapy, including 54.3% (51/94) with IA regimen, 31.9% (30/94) with DA, and 13.8% (13/94) with the other regimens. CR was observed in 74.5% (66/94) of patients, and 57.4% in the first cycle. Finally, 24.5% (23/94) underwent allogeneic hematopoietic stem cell transplantation. With a median follow-up of 7.8 (range, 0.3–35.4) months, the estimated 2-year OS rates for the cohort were 59.4% ± 9.6%. The remaining 10 patients are relapsed and refractory AML, and 50% (5/10) of them achieved CR after reinduction, with 2 patients in the first cycle. Within a median follow-up of 8.0 (range, 1.3–30.4) months, 4 patients died within half a year, and the estimated 2-year OS rates after reinduction were 24.0% ± 18.8%.

### *Ex Vivo* Chemosensitivity Results

We divided the 104 patients into sensitive (Group 4 + 5, score >60) and intermediate/resistant groups (Group 1 + 2+3, score <60) according to their optimal PharmaFlow results (highest score of the 20 treatment regimens). In all, CR was observed in 89.4% (93/104) of patients in the sensitive group and 10.6% (11/104) in the intermediate/resistant group, 71.2% in Group 5, 18.2% in Group 4, 5.8% in Group 3, 3.8% in Group 2, and 1.0% in Group 1. Compared with the intermediate/resistant group, the sensitive group had a higher CR rate (Group 4 + 5 vs. Group 1 + 2+3: 77.5% vs. 27.3%, p = 0.002) and less ELN adverse patients (22.6% vs. 63.6%, p = 0.015) (**Supplement 2**).

**Figure 1:**
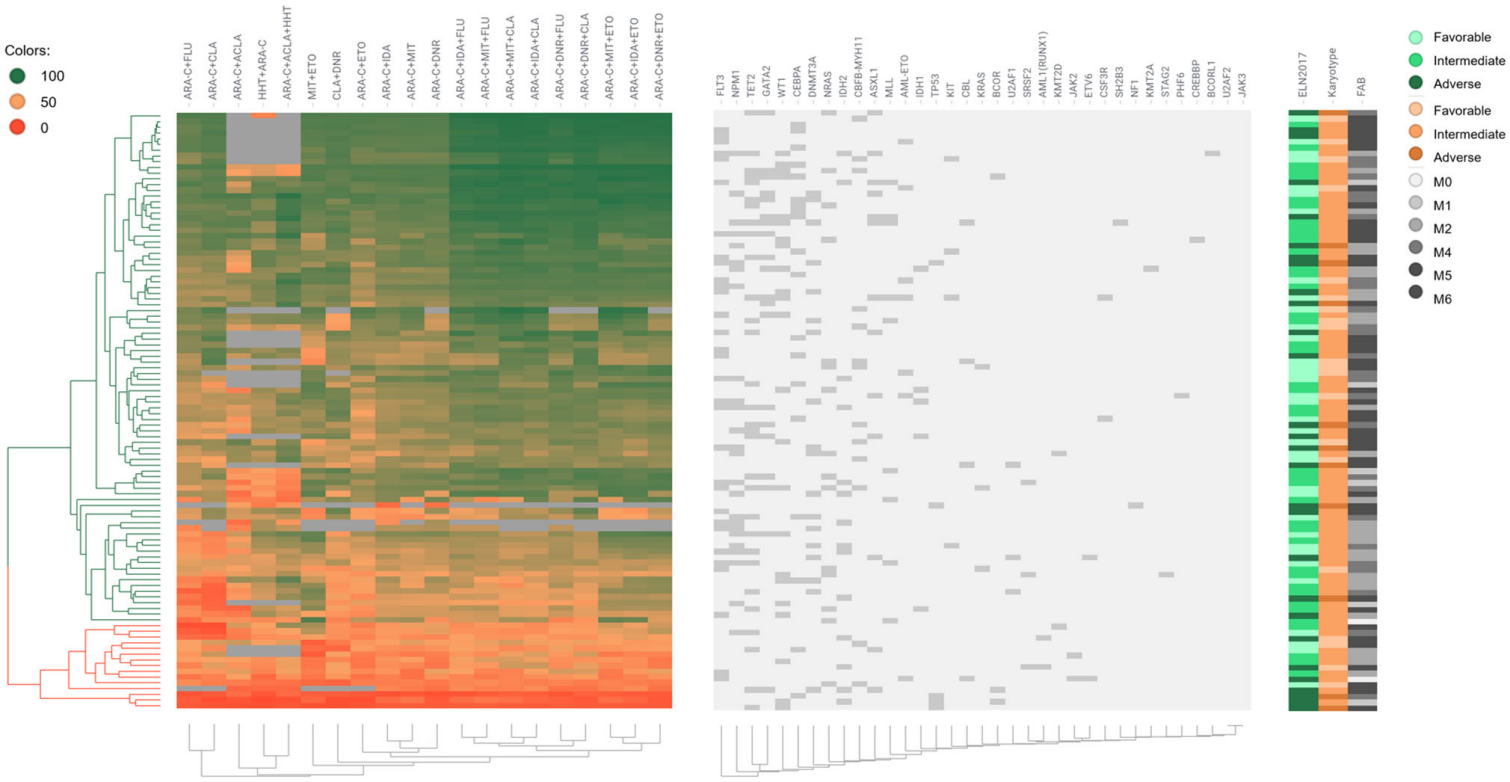
Drug sensitivity heat map of 104 AML patients. Each row represents a patient; each column represents a treatment or a stated gene; different colors represent different drug sensitivities per patient; the left side of the graph annotates the classifications of ELN 2017, karyotype, and FAB; the upper histogram shows the name of treatments and gene mutations.

We also represented the patient clinical characteristics, cytogenetic characteristics, mutations, and drug sensitivity results into a heat map (**Figure 1**). Several of the drug combinations have been applied to the study population, the heat map suggested that the 3-drug combinations *ex vivo* score better. Besides, drug sensitivity of the R/R group was significantly worse than that of the *de novo* group. The mutation frequency of NPM1 was potentially higher in sensitive patients than in resistant patients (p = 0.062). Mutation of TP53 was significantly lower in the sensitive group (p = 0.000). Drug resistance mainly occurred in the M5 subtype and adverse ELN 2017 prognosis group.

### Correlation With Clinical Response

**Figure 2:**
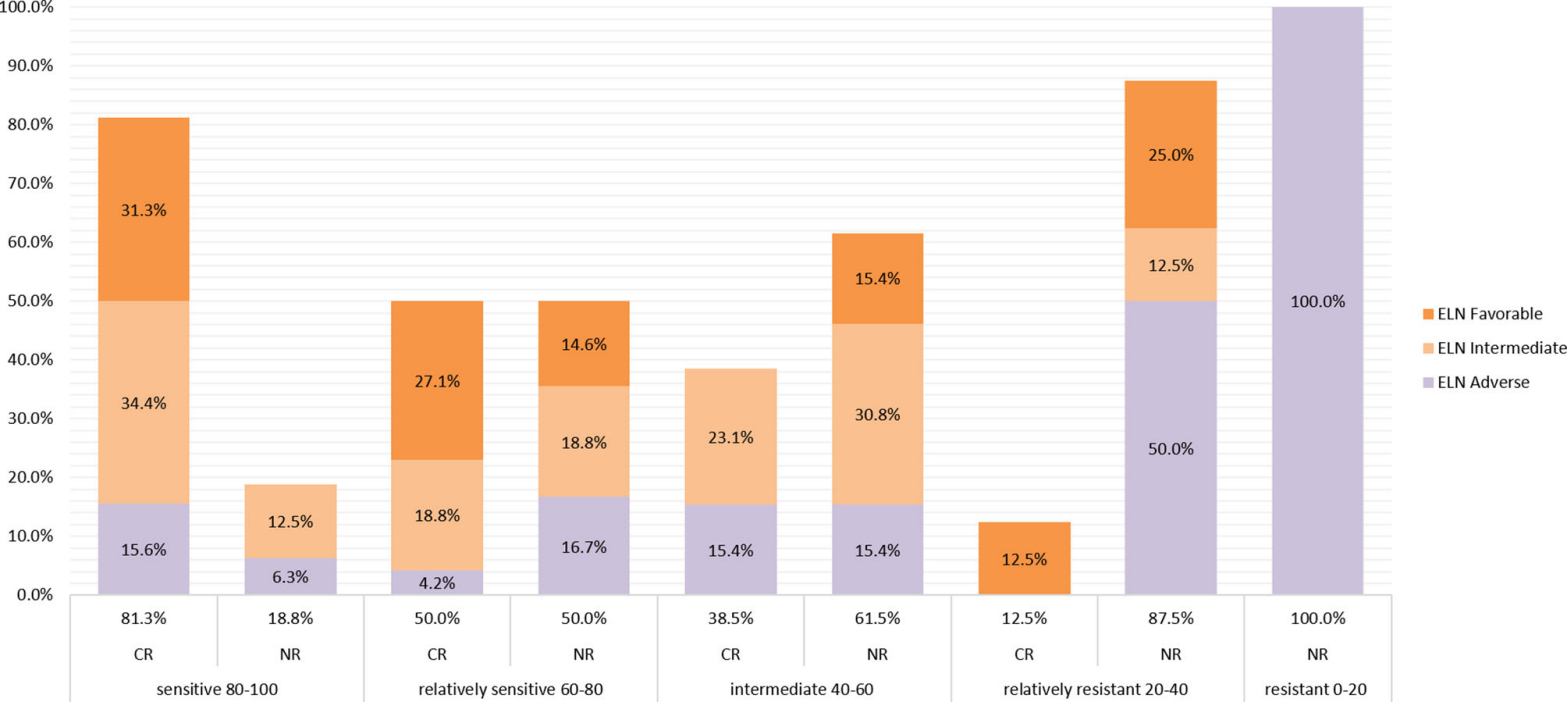
Remission rate of each sensitive group. Patients were divided into 5 groups according to the drug sensitivity results of the actual clinical induction regimen, with 32, 48, 13, 8, and 3 patients from sensitive to resistant, respectively. The clinical CR correlates with the predicted responses, which decreased from sensitive to resistant, to 81.3%, 51.0%, 38.5%, 14.3%, and 0%, respectively. The ELN 2017 classifications of CR patients and non-CR patients in different sensitivity groups are also displayed in different colors in the histogram.

All patients were divided into 5 groups according to the drug sensitivity results of the actual clinical induction regimen, with 32, 48, 13, 8, and 3 patients in Group 5 to Group 1, respectively. The clinical CR correlated with the predicted responses decreasing with drug sensitivity were 81.3%, 50.0%, 38.5%, 12.5%, and 0%, respectively (**Figure 2**). Most patients whose PharmaFlow scores were higher than 80 achieved clinical CR within the first induction regimen. Patients with low PharmaFlow scores had significantly lower clinical CR. The prediction coincidence rate of the sensitive group and the resistant group was 81.3% (Group 5) and 90.9% (Group 1 + 2), respectively.

**Figure 3:**
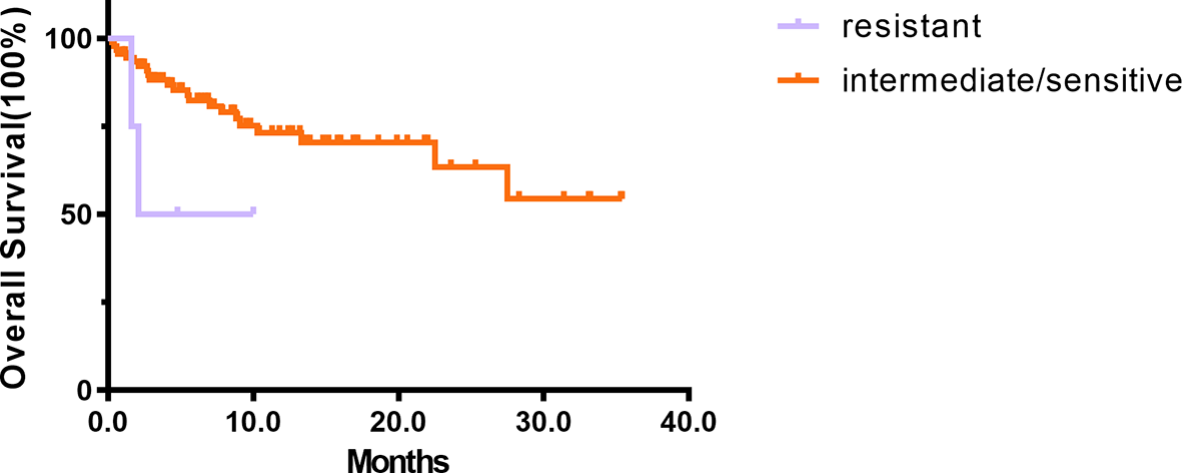
The prognosis of 94 *de novo* AML. The results of Kaplan–Meier analyses for overall survival in intermediate/sensitive (Group 3 + 4 + 5) and resistant (Group 1 + 2) patients.

The OS was shorter in the resistant patients (Group 1 + 2) than in intermediate/sensitive patients (Group 3 + 4+5) (p = 0.086) (**Figure 3**). The median OS among intermediate/sensitive (Group 3 + 4+5) and resistant (Group 1 + 2) patients was not reached and 6.0 months, respectively. In multivariable analysis, CR after the first cycle was an independent prognostic factor for OS [p = 0.001; 95% CI 0.202(0.080–0.511)], and *ex-vivo* chemosensitivity was a potential predictive factor for OS [Group 3 + 4+5 vs. Group 1 + 2 p = 0.078; 95% CI 0.696 (0.465–1.041)], **Table 2**.

**Table 2:** Univariate and multivariate analysis for overall survival.

Parameter	Univariate	Multivariate	HR (95% CI)
	p value	p value	
CR after one cycle (yes/no)	**0.001**	**0.001**	0.202 (0.080–0.511)
*Ex vivo* sensitivity (S/R)	0.086	0.078	0.696 (0.465–1.041)
Age (>35 or not)	0.065	0.098	2.392 (0.851–6.721)
Platelet count (>20 or not)	0.104	0.241	0.569 (0.222–1.461)
WBC count (>20 or not)	0.111	0.177	1.837 (0.759–4.443)
ELN risk	0.061	0.587	
Fav vs. Inter	0.069	0.378	1.749 (0.551–5.983)
Fav vs. Adv	0.018	0.320	1.898 (0.537–6.707)
Treatment (DA/IA or not)	0.359	/	/
BM blasts (>60% or not)	0.701	/	/

Factors (univariate analysis p < 0.2) underwent multivariate analysis. Significant p values are in bold.

HR, hazard ratio; CR, complete remission; S/R, intermediate/sensitive (Group 3 + 4 + 5) vs. resistant (Group 1 + 2) according to their optimal PharmaFlow results (highest score of the 20 treatments).

WBC, white blood cell; ELN, European Leukemia Net; Fav, favorable; Inter, intermediate; Adv, adverse; IA, idarubicin + cytarabine; DA, daunorubicin + cytarabine; BM, bone marrow.

### Implications for Treatment Optimization

**Figure 4:**
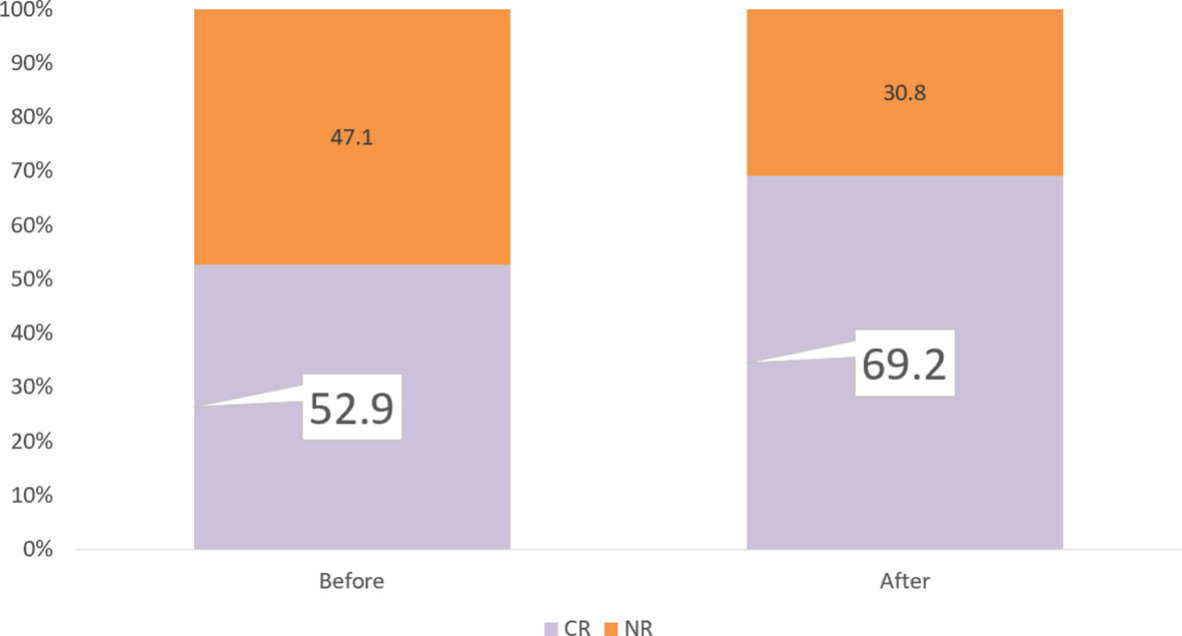
Remission rate improvement. If we select the PM test for all patients, the PM test would increase CR from 52.9% to 69.2%, with 16.5% more patients achieving CR.

The PM test predicted 81.3% CR for Group 5 Tx. In our study, 71.2% (74/104) patients had ≥1 Group 5 Tx among the total of 20 Tx. Among these 71.2% patients, the PM test achieved 81.3% CR compared with the clinical CR of 62.2%, which is a 19.1% increase. Within the remaining 29.8% (30/104) patients without any Group 5 Tx, 18.2% (19/104) had at least 1 Group 4 Tx, with an improved prediction CR rate of 50.0% compared with the clinical CR rate of 42.1%. An additional 5.8% (6/104) patients had ≥1 Group 3, without any Groups 4–5. The PharmaFlow PM test indicated an increase in CR rates from 16.7% to 38.5% and an increase of 12.8% CR in very critical patients. For Group 1 (1/104) and Group 2 (4/104), consistent with being multiresistant to most drug treatments, their predicted (0%, 12.5%) and actual clinical response (0, 25%) were poor (**Table 3**). It showed that chemotherapy individually selected by the test improved the response rates compared with the empirically selected regimens. After including all patient samples, the PM test could potentially increase CR from 52.9% to 69.2%, with 16.3% more patients achieving CR (**Figure 4**).

**Table 3:** A comparison of clinical response.

		Actual clinical response		Predicted clinical response	
Best score*^a^*	Total# (%)	CR n	CR %	CR n	CR %
Group 5	74 (71.2)	46	62.2	60	81.3
Group 4	19 (18.2)	8	42.1	9.5	50.0
Group 3	6 (5.8)	1	16.7	2	38.5
Group 2	4 (3.8)	1	25	0.5	12.5
Group 1	1 (1.0)	0	0	0	0
All	104	56	52.9	74	69.2

^a^Groups 1 to 5 were according to the optimal PharmaFlow results (highest score of the 20 treatments). The predicted CR % of each sensitive group is calculated based on the patients’ actual chemotherapy regimen and CR or not. The predicted CR % multiplied by the number of people in each group to get the predicted CR number (CR n). The difference between the predicted clinical response CR(n) and the actual clinical response CR(n) is the optimization ability of the PharmaFlow PM test.

## Discussion

In this study, we presented the *ex-vivo* chemosensitivity profile of 104 AML patients using a novel precision medicine tool—*ex vivo* PharmaFlow PM test—and analyzed the correlation with clinical response and outcomes to chemotherapy in detail. This is a considerably large multicenter *ex-vivo* drug sensitivity dataset of Chinese AML patients. Our results indicated that the individual PharmaFlow score showed a good correlation with the actual response and OS of AML patients. Optimizing treatment using the PharmaFlow PM platform is a promising method to address the problem of heterogeneity in AML and greatly improves the efficacy.

The implementation of a precision medicine approach using the PharmaFlow PM test to treat AML patients has been well documented in previous studies. In 2014, Bennett et al. used this automated flow cytometry-based platform for the first time to evaluate the *ex-vivo* pharmacology of single drugs and drug combinations in AML cells of bone marrow samples from 125 patients (21). After that, in 2019, Cuadrón et al. tested the *ex-vivo* drug activity of cytarabine, idarubicin, and their combination using the PharmaFlow platform (11). They concluded that the observed leukemic chemosensitivity of IA 3 + 7 induction showed an in-time and good correlation with the hematological response, and after validation in an external cohort, this novel *ex-vivo* test could be used to select AML patients for the 3 + 7 regimen vs. alternative schedules. In the same year, Megías et al. used the PharmaFlow platform to analyze if a subset of AML patients may respond differently to anthracyclines (idarubicin, daunorubicin, or mitoxantrone) (12). Their results showed that the patients may have heterogeneous sensitivity to different anthracyclines, and every third of the patients could benefit from this test in selecting anthracyclines. Onecha et al. analyzed the mutational profiling using next-generation sequencing in 190 AML patients and drug sensitivity using the *ex-vivo* PharmaFlow test in 74 AML patients, focused on their prediction value on clinical response and outcomes, and developed a new prognostic risk score based on both pharmacological and mutational information (16). Recently, Lin et al. used the PharmaFlow method to examine bone marrow samples from 38 Chinese AML patients with a panel of 7 single drugs and 6 combinations with cytarabine at different concentrations. Their data suggested that *ex-vivo* drug activity strongly corresponded to clinical efficacy in Chinese AML patients (17).

In our study, the test accurately predicted both clinical response and survival in patients with AML. We examined the drug sensitivity profile of newly diagnosed and relapsed AML patients in China and compared the characteristic of each subtype. We found that the drug sensitivity of the R/R group was significantly worse than that of the *de novo* group and the remission rate decreased with the decrease in predictive drug sensitivity. All these findings suggested a good correlation between the PharmaFlow platform and clinical response. Blom et al. found that *ex-vivo* chemosensitivity assays are better at predicting *in-vivo* chemotherapy resistance than sensitivity (22), while Lin et al. reported the opposite results (17). Based on our study, the PharmaFlow PM test showed a great clinical utility in predicting both sensitive and resistant patients. The sensitive prediction coincidence rate was 81.3%, and the resistant prediction coincidence rate was 90.9%. If the treatment recommendations are followed, 16.5% more patients will achieve CR in one cycle. This emphasizes the potential use of this test in predicting the response rate and selecting the optimal treatment for patients with AML. The PharmaFlow PM test can identify patients with good and poor survival (p = 0.086). The present study is one of the few studies that have reported the correlation between drug sensitivity and AML prognosis using an *ex-vivo* chemosensitivity platform. Our study indicated that as the sample size increases, a multivariate analysis may show a significant difference between drug sensitivity and OS.

The PharmaFlow PM platform can detect multiple drugs and drug combinations by using very few samples and has the characteristics of short time consumption, high accuracy, and good applicability. Despite its many advantages, this technology also has some shortcomings. Although it can accurately predict highly sensitive and resistant treatment regimens, it has a poor clinical value for regimens in the intermediate resistance. Targeted drugs such as venetoclax, gilteritinib, dasatinib, and bortezomib are not yet included, although they are being developed.

One important limitation is the non-prospective design; the induction treatments were not completely unified, which might have caused a deviation in the prognostic analysis. Second, the non-prospective design makes it difficult to dynamically observe the drug sensitivity of the same patient before treatment and at the time of relapse. Thirdly, due to the limitation of the number of cases, only a few parameters were included in the multivariable analyses. Further steps should be taken to streamline the process for clinical use and to evaluate the clinical benefit of the test platform. First, large prospective randomized controlled studies comparing standard versus assay-directed therapy with an endpoint of CR or OS are required before routine clinical utilization of these assays. Second, more precise analysis is required to explore the prediction differences of this technology in different karyotypes and molecular mutations, especially TP53, FLT3-ITD, and MLL mutations that make CR difficult to achieve, to improve the prognosis of the high-risk group. Finally, collecting more samples to build a database based on Chinese AML patients is necessary for more accurate predictions.

## Conclusions

To summarize, the *ex-vivo* PharmaFlow PM test is a rapid and efficient technology worthy of clinical promotion, with good clinical application value in AML patients, which is helpful for the prediction of chemotherapy efficacy and outcomes.

## Data Availability Statement

The raw data supporting the conclusions of this article will be made available by the authors, without undue reservation.

## Ethics Statement

The studies involving human participants were reviewed and approved by the Research Ethics Committee of the First Affiliated Hospital, College of Medicine, Zhejiang University, Qilu Hospital of Shandong University, and Shanghai General Hospital. Written informed consent to participate in this study was provided by the participants’ legal guardian/next of kin.

## Author Contributions

H-HZ and YZ drafted the manuscript and contributed to the final draft. C-WW performed the experiments and analyzed and interpreted data. Others collected the data of the manuscript. All authors contributed to the article and approved the submitted version.

## Funding

This study was funded by the Natural Science Foundation of Shandong Province (ZR2020KH016) to C-YJ, the Leading Innovative and Entrepreneur Team Introduction Program of Zhejiang (2020R01006 and 2019R01001) to H-HZ, the Natural Science Foundation of China (81820108004 and 82170144) to JJ, and the key research and development program of Zhejiang (2021C03123) to JJ.

## Conflict of Interest

Author C-WW was employed by Hosea Medical Technology (Beijing) Co., Ltd. Author JB is employed by Vivia Biotech.

The remaining authors declare that the research was conducted in the absence of any commercial or financial relationships that could be construed as a potential conflict of interest.

## Publisher’s Note

All claims expressed in this article are solely those of the authors and do not necessarily represent those of their affiliated organizations, or those of the publisher, the editors and the reviewers. Any product that may be evaluated in this article, or claim that may be made by its manufacturer, is not guaranteed or endorsed by the publisher.
