# Supplementary material for: Ex Vivo Chemosensitivity Profiling of Acute Myeloid Leukemia and Its Correlation With Clinical Response and Outcome to Chemotherapy
Source: Front Oncol. 2022 Jan 5;11:793773. doi: 10.3389/fonc.2021.793773 (PMC8767104; doi:10.3389/fonc.2021.793773)
Supplement: Supplementary file 1 [file Table_1.docx]

**S1 Treatments panel**

| **Number** | **abbreviation** | **Full name of the treatments** |
| --- | --- | --- |
| 1 | IDA + Ara-C | Idarubicin + Cytarabine |
| 2 | DNR + Ara-C | Daunorubicin + Cytarabine |
| 3 | MIT + Ara-C | Mitoxantrone + Cytarabine |
| 4 | ACLA + Ara-C | Aclarubicin + Cytarabine |
| 5 | HHT + Ara-C | Homoharringtonine + Cytarabine |
| 6 | Ara-C + Flu | Cytarabine + Fludarabine |
| 7 | Ara-C + CLA | Cytarabine + Cladribine |
| 8 | Ara-C + ETO | Cytarabine + Etoposide |
| 9 | DNR + CLA | Daunorubicin + Cladribine |
| 10 | MIT + ETO | Mitoxantrone + Etoposide |
| 11 | ACLA + Ara-C +HHT | Aclarubicin + Cytarabine+ Homoharringtonine |
| 12 | Ara-C + IDA + FLu | Cytarabine+ Idarubicin + Fludarabine |
| 13 | Ara-C + DNR + FLu | Cytarabine+ Daunorubicin + Fludarabine |
| 14 | Ara-C + MIT + FLu | Cytarabine+ Mitoxantrone + Fludarabine |
| 15 | Ara-C + IDA + CLA | Cytarabine+ Idarubicin + Cladribine |
| 16 | Ara-C + DNR + CLA | Cytarabine+ Daunorubicin + Cladribine |
| 17 | Ara-C + MIT + CLA | Cytarabine+ Mitoxantrone + Cladribine |
| 18 | Ara-C + IDA + ETO | Cytarabine+ Idarubicin + Etoposide |
| 19 | Ara-C + DNR + ETO | Cytarabine+ Daunorubicin + Etoposide |
| 20 | Ara-C + MIT + ETO | Cytarabine+ Mitoxantrone + Etoposide |

**S2 General characteristics of 104 AML patients based on** **ex vivo Chemosensitivity**

| Group | Sensitive | Intermediate/Resistant | P |
| --- | --- | --- | --- |
| cases | 93 | 11 |  |
| Gender(F/M) | 45/48 | 5/6 | 0.854 |
| Age, years | 43.0(14-64) | 47.0(13-62) | 0.692 |
| WBC, 10^9/L | 17.2(0.15-305.3) | 3.64 (0.53-42.9) | 0.010 |
| HB, g/L | 83(32-162) | 82(49-114) | 0.590 |
| PLT, 10^9/L | 35(5-639) | 32.0(6-212) | 0.845 |
| Blasts, % | 74(18-95) | 45(20-90) | 0.085 |
| FAB |  |  | 0.021 |
| M0 | 1(1.1%) | 1(9.1%) |  |
| M1 | 5(5.4%) | 1(9.1%) |  |
| M2 | 25(26.9%) | 3(27.3%) |  |
| M4 | 25(26.9%) | 1(9.1%) |  |
| M5 | 37(39.8%) | 4(36.4%) |  |
| M6 | 0(0.0%) | 1(9.1%) |  |
| Mutations |  |  |  |
| FLT3^*^ | 29(31.2%) | 2(18.2%) | 0.499 |
| TET2 | 27(29.0%) | 3(27.3%) | 1.000 |
| NPM1 | 25(26.9%) | 0(0.0%) | 0.062 |
| WT1 | 20(21.5%) | 3(27.3%) | 0.704 |
| GATA2 | 19(20.4%) | 1(9.1%) | 0.686 |
| DNMT3A | 17(18.3%) | 1(9.1%) | 0.685 |
| NRAS | 14(15.1%) | 1(9.1%) | 1.000 |
| IDH2 | 11(11.8%) | 3(27.2%) | 0.166 |
| TP53 | 1(1.1%) | 4(36.4%) | **0.000** |
| ELN2017 |  |  | **0.015** |
| Favorable | 33(35.5%) | 2(18.2%) |  |
| Intermediate | 39(41.9%) | 2(18.2%) |  |
| Adverse | 21(22.6%) | 7(63.6%) |  |
| CR/NR | 77.5%/22.5% | 27.3%/72.7% | **0.002** |
| CR after 1 cycle | 58.1% | 9.1% |  |

Significant P values are in bold.

Sensitive (Group 4+5, score >60) and intermediate/resistant group (Group 1+2+3, score <60) according to their optimal PharmaFlow results (highest score of the 20 treatments).

WBC, white blood cell count; Hb, Hemoglobin; PLT, platelet count; FAB, morphology according to French–American–British classification; ELN, European Leukemia Net; CR, complete remission.

*: FLT3-ITD and FLT3-TKD all included.
